# Geographic availability of and physical accessibility to tuberculosis diagnostic tests in Ghana: a cross-sectional survey

**DOI:** 10.1186/s12913-023-09755-3

**Published:** 2023-07-14

**Authors:** Desmond Kuupiel, Benjamin S. N. Cheabu, Peter Yeboah, James Duah, Joseph K. Addae, Ignatius T. Ako-Nnubeng, Francis A. Osei, Shamsu-Deen Ziblim, Gugu G. Mchunu, Julian D. Pillay, Vitalis Bawontuo

**Affiliations:** 1 Discipline of Public Health Medicine, School of Nursing and Public Health, University of KwaZulu-Natal, Durban, 4001 South Africa; 2 Faculty of Health Sciences, Durban University of Technology, Ritson Campus, Durban, 4001 South Africa; 3 Faculty of Health Sciences, Health Quality Programs, Queen’s University, Kingston, K7L3N6 Canada; 4 Christian Health Association of Ghana, # 21 Jubilee Wells Street Labadi, Accra, Ghana; 5 Department of Population and Reproductive Health, School of Public Health, University of Development Studies, Tamale, Ghana; 6 Department of Health Services Management and Administration, School of Business, SD Dombo University of Business and Integrated Development Studies (SDD-UBIDS), Wa, Ghana

**Keywords:** Tuberculosis, Diagnosis, Availability, Physical Accessibility, Ghana

## Abstract

**Background:**

In Ghana, tuberculosis (TB) case detection is low (< 34%). Existing scientific evidence suggest access to TB diagnostic tests play an essential role in TB case detection, yet little has been scientifically documented on it in Ghana. This study, therefore, sought to map TB diagnosis sites, and describe the geographic availability and physical accessibility to TB diagnosis in six regions of Ghana to inform scale-up and future placement of TB diagnostic tests.

**Methods:**

We assembled the geolocation and attribute data of all health facilities offering TB diagnosis in Upper West Region (UWR), Upper East Region (UER), Ahafo, North-East, Northern, and Savannah regions. QGIS was employed to estimate the distance and travel time to TB diagnosis sites within regions. Travel time estimates were based on assumed motorised tricycle speed of 20 km (km)/hour.

**Results:**

Of the total 1584 health facilities in the six regions, 86 (5.4%) facilities were providing TB diagnostic testing services. This 86 TB diagnosis sites comprised 56 (65%) microscopy sites, 23 (27%) both microscopy and GeneXpert sites, and 7 (8%) GeneXpert only sites (8%). Of the 86 diagnosis sites, 40 (46%) were in the UER, follow by Northern Region with 16 (19%), 12 (14%) in UWR, 9 (10%) in Ahafo Region, 5 (6%) in North East, and 4 (5%) in Savannah Region. The overall estimated mean distance and travel time to the nearest TB diagnosis site was 23.3 ± 13.8 km and 67.6 ± 42.6 min respectively. Savannah Region recorded the longest estimated mean distance and travel time with 36.1 ± 14.6 km and 108.3 ± 43.9 min, whilst UER recorded the shortest with 10.2 ± 5.8 km and 29.1 ± 17.4 min. Based on a 10 km buffer of settlement areas, an estimated 75 additional TB diagnosis sites will be needed to improve access to TB diagnosis services across the six regions.

**Conclusion:**

This study highlights limited availability of TB diagnosis sites and poor physical accessibility to TB diagnosis sites across five out of the six regions. Targeted implementation of additional TB diagnosis sites is needed to reduce travel distances to ≤ 10 km.

**Supplementary Information:**

The online version contains supplementary material available at 10.1186/s12913-023-09755-3.

## Background

Tuberculosis (TB) is caused by Mycobacterium tuberculosis and mostly affects the lungs. TB is spread through the air when people with pulmonary TB cough, sneeze or spit. Globally, TB is the 13th leading cause of death and the second leading infectious killer after COVID-19 (above HIV/AIDS). In 2021, the World Health Organization (WHO) estimated that about 10.6 million people fell ill with TB globally. That is, 6 million men, 3.4 million women and 1.2 million children [1]. Tuberculosis is found in all countries and age groups, but most of the people who fall ill with TB live in low- and-middle-income countries (LMICs) including Ghana [1].

In 2022, the Centers for Disease Control and Prevention ranked TB as the seventh top cause of death in Ghana responsible for about 4.91% of all deaths [2, 3]. Despite this, research shows TB case detection in the country is about 34% which is lower than the WHO estimated target of 80% [4–6]. To increase TB case detection, the Government of Ghana (GOG) together with her local and international partners, including The Global Fund have made several efforts through investments in laboratories, equipment, and supplies [3]. Aside from the retooling of laboratories across the country's hospitals, as well as intensified TB case findings; procurement and distribution of GeneXpert MTB/RIF test for some health facilities, the National TB Control Program has in addition implemented what is known as the "spokes" and "hub" system which requires that Civil Society Organisations (CSOs) operating in hard-to-reach areas without a testing site should submit their sputum samples to a designated point (spokes) for later transportation to the testing facility (hubs). Despite these interventions, there remain some diagnostic difficulties hence, an estimated 31,326 people with TB were missing out of the 44,0000 estimated people who developed TB in 2021 [3]. Reasons contributing to this huge number of missing TB cases may be multifaceted including poor access to TB diagnostic services [5, 6].

A recent study indicated that there is poor geographic accessibility to TB testing services at point-of-care in Ghana [7]. The study found that the majority (62%) of the population resident in the study area travel more than 10 km over several hours to access a health facility providing TB diagnostic services [7]. Previous studies have demonstrated the impact of access to TB services on TB control programs in sub-Saharan Africa. For instance, in Ethiopia, TB case notification rates were found to be higher in areas where people had good access to diagnostic and treatment facilities [8]. Also, another study that involved high TB/HIV burden African countries demonstrated that travel time was associated with delays in patients returning for care [9, 10]. To this end, there is the need to expand access to TB health services such as TB diagnosis closer to where people live and work. The expansion of TB diagnosis services potentially can increase TB case detection in Ghana, and enable it to meet its global targets as stipulated by the End TB Strategy and sustainable development goal 3.3 (By 2030, end the epidemics of TB) amidst the thread of COVID-19 pandemic and economic constraints. For this reason, it is prudent to identify priority areas for targeted TB diagnostic services improvement due to limited resources using Geographic Information Systems (GIS) and the spatial statistical packages available for analysis of epidemiological data, in GIS.

A GIS is a computer-based system where data that are linked to a geographic place (known as geo-referenced data) can be entered, managed, manipulated, analysed and displayed [11]. GIS platforms are ideal for bringing together disease-specific information and analysing it in relation to population settlements, surrounding social and health services, and the natural environment[12]. They are ideal for analysing epidemiological data, revealing spatial trends and interrelationships that would be difficult to detect in tabular format [12]. Furthermore, GIS allows policymakers to easily visualize problems about existing health and social services as well as the natural environment, allowing them to target resources more effectively [12]. GIS has been used in many ways to inform TB control programs in several countries such as South Africa [13], Ethiopia [8], Brazil [14], and China [15].

In Ghana, the application of GIS to inform the placement of TB control services including diagnostic services would be a novelty. Although a previous study in Ghana used GIS to estimate geographical access to TB diagnostic services, it focused on only one out of the sixteen regions in the country [7]. Therefore, this study aimed to employ GIS to map TB diagnosis sites (Microscopy and GeneXpert), evaluate the physical accessibility (distance and travel time) to existing TB diagnosis sites, and identify settlement locations with poor access to TB diagnosis sites as well as sites for future placement of TB diagnostic services in six regions (Upper West, Upper East, Northern, North-East, Ahafo and Savannah regions) of Ghana.

## Methods

### Design and data sources

**Fig. 1:**
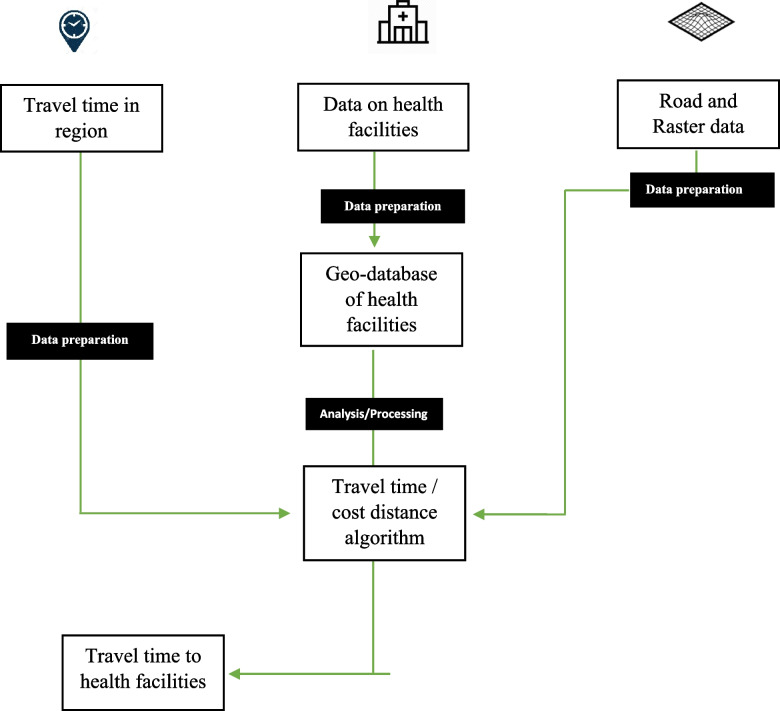
Diagram showing the flow for estimating accessibility to health facilities in in Ahafo, Upper West, North-East, Northern, Savannah, and Upper East Regions of Ghana

Figure 1 schematically depicts the various data and modelling components used to achieve the study's aim. The study was a cross-sectional survey designed to source data from all six participating regions using a structured questionnaire. The data included names of regions, districts, health facility names, health facility types, ownership, latitude, longitude, availability of TB diagnostic testing, and type of diagnostic (Microscopy, GeneXpert, or both). The geo-locational data of all health facilities and associated attribute data were obtained from the respective Regional Health Directorates of the six participating regions. The geo-located data of the health facilities were collected by trained personnel of the Ghana Health Service using calibrated global positioning system devices (Garmin—Handheld GPSMAP). The geolocation and attribute data of health facilities was collected between September and November 2021.

The geolocation and attribute data of health facilities, which were in tabular format, were imported into QGIS software and transformed into shapefiles. Other datasets included topographical data such as road networks, rivers, digital elevation models, and shapefile files for the regions. The topographical dataset in the field was obtained to help estimate travel time. To ensure data accuracy, the topographical data obtained was juxtaposed with topographical data obtained from the University of Ghana's Remote Sensing and Geographical Information System Laboratory. Travel time was estimated via road, paths and tracks using a motorised tricycle popular called "Pragyia" or "Mahama can do" or "motor king" because it was found to be the most used transportation system in all the participating regions. The process involved recalibrating travel time per pixel (10 m by 10 m grid) for both roads and paths. This recalibration was used to estimate travel time from all locations to TB testing sites in each region. Ghana Regional and District boundaries shapefiles were obtained from ArcGIS Hub (USAID Ghana). The WGS Zone 30 North coordinate system was applied to the dataset since Ghana falls within this zone. This application accurately mapped all the latitudes and longitude to their relative locations on the earth.

### Model for estimating travel time

The model used to estimate the travel time for this study has been explained in detail in our previous studies [7, 16–19]. The QGIS application was used to estimate the travel time in this research and key algorithms like the raster to polygon conversion and cost distance were used in the analysis. The cost distance model calculates the shortest time to a source based on a cost dataset; hence, it was used to determine distance. To start with, a cost surface algorithm was designed with these parameters: a grid cell of size 10 m was assigned to the spatial features, and values were then assigned to the predetermined grids (Cost values i.e. time to travel with a speed of 20 km / hour per grid). The essence of this design was to grant the user control over travel time estimation. The dataset was divided into grids or cells and as a strategy to determine travel time, these grids or cells were leveraged to calculate how much users should travel and the equivalent of that in time. In essence, to travel a 10 m grid of roads, the travel time on roads and the medium of travel is considered and this eventually determined the value of time for the 10 m grid of road. Based on this logic, roads were assigned low values because traveling by road is faster than traveling via paths. Assigning values to spatial features was done via the vector to raster conversion tool. Cost distance requires the cost surface dataset and the focus points, that is, health facilities offering TB diagnosis services. The output is a map showing shortest travel time (cell by cell) from any cell on the map to any health facilities in the region. The optimal cell size is needed to be determined because it impacts the results of the study. The cell size must be small enough to capture the details on the image and it should not be too large to affect the efficiency of processing the algorithm or model [7].

The common mode of travel in the regions, which are predominantly rural, is a motorised tricycle properly known as “Pragyia” or “Mahama can do” or "motor king" and the travel speed is assumed to be 20 km/h. This served as a guide for determining travel time in the participating regions. The travel time calculation leverage on the cell size of 10 m and synchronised the units with the assumed speed of 20 km/h was converted to meters. To estimate how many minutes, it takes to travel 10 m per cell, a new unit of meters per second was established. For travel via motorised tricycle, the cell size was set at 0.3 min (18 s) to cover a cell. This algorithm was applied to the regional shapefile and road network.

### Outcome measures

This study's first outcome was availability of TB diagnosis facilities in the six regions. This availability (are there TB diagnostic resources in the facility) was measured using "Yes" and "No" and described using proportions. The second outcome was physical accessibility (distance) to TB testing sites in the six participating regions. Physical accessibility was measured as 0-10 km = good physical access, and > 10 km = poor physical access [7]. Considering ≤ 10 km as good physical accessibility is arbitrary, but evidence shows that access to healthcare beyond 10 km is associated with higher risks of adverse health outcomes [20]. Also, categorising physical accessibility using travel time would have been useful but, travel time depends on the mode of transportation option and the route hence our choice to base our categorisation of physical accessibility on distance. The third outcome of this study was to identify high priority locations for TB testing implementation (where to place TB diagnostic tests to optimize access to testing) in the six regions. This outcome was achieved using geographical models and the application of remote sensing through satellite imagery analysis. The application of remote sensing through the satellite imagery analysis allowed us to observe locations that had settlements and thus, accurately identified where to place TB diagnostic tests to optimize access to testing based on a 10 km buffer in each region.

## Results

### Geographic distribution of health facilities across the six regions

**Fig. 2:**
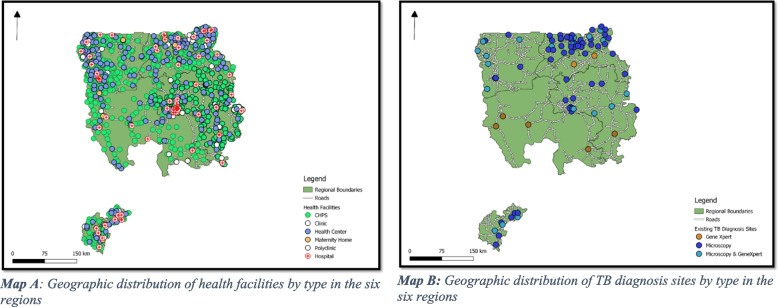
Geographic distribution of health facilities and TB diagnosis sites in Ahafo, Upper West, North-East, Savannah, Northern, and Upper East Regions of Ghana

A total of 1584 health facilities were identified in the six regions of which 438 (27.7%) were in the Upper West Region, 392 (24.7%) in Upper East Region, 376 (23.7%) in Northern Region, 176 (11.1%) in Savannah Region, 113 (7.1%) in Ahafo Region, and 89 (5.6%) in North East Region. The 1584 health facilities comprised 1116 (70.5%) Community Health Planning and Services (CHPS), 267 (16.9%) health centres, 101 (6.4%) clinics, 79 (5.0%) hospitals, 12 (0.8%) maternity homes, and 9 (0.6%) polyclinics (Fig. 2). Of the 1584 health facilities, 1416 (89.4%) were owned by the Government of Ghana, 88 (5.6%) were private, 74 (4.7%) were owned by the Christian Health Association of Ghana (CHAG), 5 (0.3%) were Quasi-Government, and 1 (0.1%) was owned by a mining company (Fig. 2, Map A).

### Geographic availability of TB diagnosis sites across the six regions

Of the 1584 facilities in the six regions, 86 (5.4%) were providing TB diagnosis services. Of the 86 TB diagnosis sites in the six regions, 56 (65.1%) were Microscopy only sites, 20 (23.3%) were both Microscopy and GeneXpert sites, and 10 (11.6%) GeneXpert only sites (Fig. 2, Map B). Forty (46.5%) of these facilities were in the Upper East Region, 16 (18.6%) in Northern Region, 12 (14.0%) in Upper West Region, 9 (10.5%) in Ahafo Region, 5 (5.8% in North East, and 4 (4.7%) in Savannah Region (Supplementary file 1).

### Physical accessibility to TB diagnostic testing sites

#### Distance

**Fig. 3:**
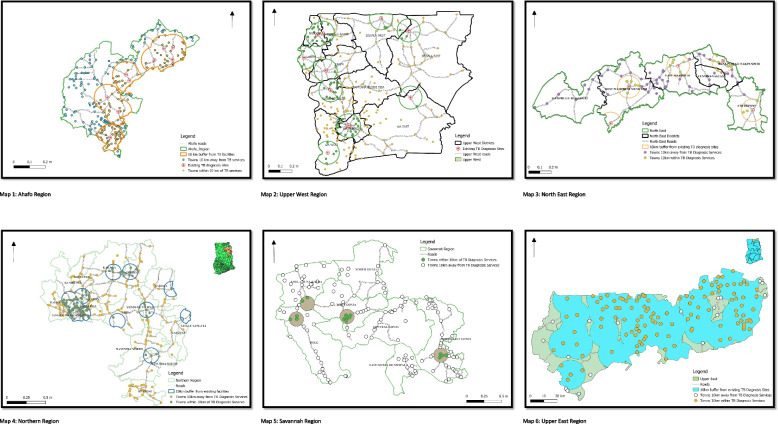
Map showing geographic locations of towns and their proximity to a health facility providing TB diagnostic service within 10 km buffer in Ahafo, Upper West, North East, Savannah, Northern, and Upper East Regions of Ghana

A total of 1196 towns were identified in all the six regions via Google Map of which 479 (40%) were located within 10 km proximity to a TB diagnosis site. Based on a 10 km buffer around TB diagnosis sites, 50% (139/278) of towns in the Ahafo region, 28% (77/278) of towns in UWR, 32% (29/90) of towns in North East Region, 38% (95/253) of towns in Northern Region, 10% (16/155) of towns in Savannah Region, and 87% (123/142) of town in UER were within 10 km proximity to a TB diagnosis site (Fig. 3). For the six regions, the mean (SD) distance to a TB diagnosis site was approximately 23 (14) km. The mean (SD) distance to a TB diagnosis site was 24 (20.6) km in the Ahafo Region, 22 (14) km in UWR, 27 (17) km in North East Region, 21 (11) km in Northern Region, 36 (15) km in Savannah Region, and 10 (6) km in Upper East Region.

### Travel time

Supplementary file 2 presents visual presentations of the travel time in all the six participated region. The estimated travel time to access a TB diagnosis site for the six regions was approximately 87 (43) mins using “Pragyia” (a motorised tricycle) at an assumed speed of 20 km/h. The mean (SD) travel time to a TB diagnosis site was 59 (69) mins in the Ahafo Region, 65 (42) mins in UWR, 81(52) mins in North East Region, 64 (32) mins in Northern Region, 108 (44) mins in Savannah Region, and 29 (17) mins in Upper East Region. Sagnarigu district in Northern Region recorded the shortest mean distance (4.7 km) whilst Mamprugu Moagduri district in the North East Region recorded the longest mean distance (66 km) as well as the longest mean travel time (198 min) to the nearest TB diagnosis site. However, Tempane district in the Upper East Region recorded the shortest mean travel time (10.8 min) to the nearest TB diagnosis site (Table 1).

**Table 1:** Estimates of mean distance (km) and travel time (mins) per district from all locations to the nearest TB diagnosis site in Ahafo, Upper West, North East, Savannah, Northern, and Upper East Regions of Ghana

Region	District	Mean (SD) distance (km)	Mean (SD) travel time (mins) with a motorised tricycle at 20 km/h assumed speed
Ahafo Region	Asunafo North	33 (18)	90 (86)
	Asunafo South	20 (16)	60 (57)
	Asutifi North	32 (25)	80 (76)
	Asutifi South	20 (19.8)	61 (59.5)
	Tano North	19 (21)	57 (63)
	Tano South	20 (24)	60 (71)
Upper West Region	Nandom	12 (16.3)	36 (48.8)
	Lambussie-Karni	12.8 (14.7)	83 (44.1)
	Lawra	13.7 (13)	41 (39.1)
	Jirapa	19.3 (10.9)	58 (32.7)
	Nadowli-Kaleo	16.4 (11.9)	49 (35.6)
	Wa West	21.9 (11)	65.7 (33)
	Wa Municipal	10.1 (5.2)	30 (15.7)
	Wa East	35.3 (15.1)	106 (45.3)
	Sissala East	35.8 (23.6)	108 (70.8)
	Sissala West	24.4 (17.4)	73 (52.2)
North-East	Chereponi	16.6 (19.5)	50 (58.5)
	Bunkpurugu-Nakpanduri	19.6 (23.2)	59 (69.7)
	Yunyoo-Nasuan	24.5 (4.9)	73 (14.6)
	East Mamprusi	16.4 (15.8)	49 (47.4)
	West Mamprusi	17.9 (16.6)	54 (49.9)
	Mamprugu Moagduri	66 (24.5)	198 (73.4)
Northern Region	Gushegu	20.6 (11.2)	62 (33.7)
	Karaga	22 (11.7)	66 (35)
	Kpandai	37.8 (12.8)	113 (38.3)
	Kumbungu	42.2 (17.7)	127 (52.9)
	Mion	21.2 (8.5)	64 (25.4)
	Nanton	13.2 (5.4)	40 (16.1)
	Nanumba North	24.8 (12.1)	74 (36.4)
	Nanumba South	18.9 (11.9)	57 (35.6)
	Saboba	17.8 (9.8)	54 (29.5)
	Sagnarigu	4.7 (2.5)	14 (7.4)
	Savelugu	19.9 (11.9)	60 (35.9)
	Tamale	9.8 (9.9)	29 (29.9)
	Tatale Sangule	25.4 (15.9)	76 (47.6)
	Tolon	23.1 (12.9)	69 (38.7)
	Yendi	14.7 (6.9)	44 (20.7)
	Zabzugu	23.3 (6.7)	70 (20.2)
Savannah Region	Bole	31.1 (16.9)	93 (50.8)
	Central Gonja	42.4 (14.4)	127 (43.1)
	East Gonja	50.4 (14.1)	151 (42.1)
	North-East Gonja	23.9 (14.1)	72 (42.4)
	North Gonja	52.9 (16.6)	159 (49.8)
	Sawla-Tuna-Kalba	30.4 (15.9)	91 (47.9)
	West Gonja	21.6 (10.4)	65 (31.2)
Upper East Region	Binduri	12.9 (3.6)	39 (10.7)
	Bolga East	5.1 (2.8)	15 (8.4)
	Bongo	8.3 (6.9)	25 (20.6)
	Builsa North	10.4 (4.9)	31 (14.7)
	Builsa south	16.3 (8.9)	49 (26.7)
	Garu	8.7 (5.9)	26 (17.8)
	Kassena-Nankana Municipal	8.1 (4.3)	24 (12.9)
	Kassena-Nankana West	12 (7.7)	36 (23)
	Nabdam	6.2 (4.1)	19 (12.2)
	Pusiga	9.8 (7.9)	30 (23.6)
	Talensi	8.6 (7.3)	26 (21.9)
	Tempane	33 (19.3)	10.8 (6.4)

### Where to place TB diagnostic tests to optimize access to testing

To optimize access to TB diagnostic tests in the six regions, this study’s analysis suggested that at least 75 additional TB diagnosis sites will be needed across the six regions. Based on a 10 km buffering of settlement areas in each of the six regions, the analysis showed that 10 TB diagnosis sites will be needed in the Ahafo Region, 29 in Upper West Region, 13 in North East Region, 8 in Northern Region, and 15 in Savannah Region. Table 2 presents the health facilities identified to be the most appropriate (geographically) sites for future implementation of these proposed 75 TB diagnosis sites.

**Table 2:** Where to place TB diagnostic tests to optimize testing per region (based on 10 km buffering of settlement areas in Ahafo, Upper West, North East, Savannah, Northern, and Upper East Regions of Ghana

Ahafo Region (*N* = 10)	Upper West Region (*N* = 29)	North East Region (*N* = 13)	Northern Region (*N* = 8)	Savannah Region (*N* = 15)
Yamfo Health Centre	Babile Health Centre	Namangu CHPS	Wantugu Health Centre	Church of God Medical Centre
Ampenkro Health Centre	Happah CHPS	Gbankurugu CHPS	Lingbunga CHPS	Tinga Health Centre
Gyasikrom CHPS	Karni Central Clinic	Montana CHPS	Diare Health Centre	Kporibayiri CHPS
Aweawoho-Manhyia CHPS	Fian Health Centre	Kpasenkpe Health Centre	Katani CHPS	Soma CHPS
Tweneboa CHPS	Issa Health Centre	St. Patrick Health Centre	Bumbong Health Centre	Gingabuo Health Centre
Duruwaakrom CHPS	Dakyie CHPS Zone	Wungu CHPS	Kpanashei CHPS	Achulokura CHPS
Gambia health centre	Gbankor CHPS	Yikpabongo CHPS	Bincheratanga Health Centre	Mole CHPS
Subreso CHPS	Kuzie CHPS	Yamah CHPS	Keitejeli CHPS	Tari Health Centre
A new health facility with TB diagnosis in Kofi Manukrom community to serve the residents of Kofi Manukrom, Kokofu, Dwenase, Beposo, Kwasu Agya, Ntotorso, Dwenase, Kwawbukrom, Gyamfikrom, Bediakokrom, and Mayeden	Dorimon Health Centre	Yagaba Health Centre		Busunu Health Centre
A new health facility with TB diagnosis in Nyamebekyere community to serve the residents of Nyamebekyere, Yaw Owusukrom, Wamahinso, Goamu, Kwahu, Bojampa No.2, Adukojo, Kensere, Kenyase No 3, and Goaso Small	Vieri CHPS	Temaa CHPS		Buipe Polyclinic
	Poyentanga Health Centre	Kubori Health Centre		Yapei Health Centre
	Nyaminjan CHPS	Fame (Yiezesi) Health Centre		Abrumase Health Clinic
	Tuosah CHPS Compound	Shelinvoya CHPS		Buma Clinic
	Kpalwugu CHPS Compound			A new health facility with TB diagnosis in Fuu community to serve the people residing in Fuu, Banchagu, Gbung, Libi, Lakobenyale, Tantuya, and Zuyili
	Kunyebin CHPS Compound			A new health facility with TB diagnosis at Kabieso, community to serve the people residing in Kabieso, Sabonjida, Loloto, Chambuligu, Okyerepe, and Dokugere
	Bugubelle CHPS			
	Dolibizon CHPS			
	Dasima CHPS			
	Kansec Clinic			
	Kunchogu Health Centre			
	Ducie CHPS Compound			
	Gurungu Health Centre			
	Dandafuro CHPS			
	Sankana CHPS			
	Jonfiang CHPS Compound			
	Danyawkura CHPS Compound			
	Banonyiri CHPS			
	Samoa Health Centre			
	Konchuri CHPS			

## Discussion

We described the geographic availability of and physical accessibility to TB diagnostic tests to inform scale-up and future placement of TB diagnostic services in the Upper West, Upper East, Northern, North-East, Ahafo, and Savannah regions of Ghana. The results showed that less than 6% of the health facilities in the six regions were providing TB diagnosis services (GeneXpert only, Microscopy only, or both). Moreso, the majority of the available TB diagnosis sites were microscopy sites instead of GeneXpert sites – the preferred choice for TB diagnosis in Ghana. It is worth mentioning that the total population of these six regions was 6,390,547 based on the recent population and housing census report [21]. As such, the 86 diagnosis sites identified by this study translate to a ratio of one facility per 74,309 population. Although not all patients visiting a health facility will necessarily require TB testing, this ratio of health facility to populations may have dire implications for TB control program in Ghana if not addressed. For instance, the likely pressure on the few TB diagnosis sites may result in a frequent breakdown of equipment, stockout of cartridges, reagents, and other resources. Moreover, the likely increase of workload on laboratory professionals in these TB diagnosis sites over and above the afore-mentioned possible challenges might increase test turnaround time, delay TB case detection, and consequently results in poor TB treatment monitoring in these regions.

This study also found a regional variation in terms of the count of TB diagnosis sites in the participating regions. Regional population [21] and TB burden [22] differentials or inadequate availability of diagnostic equipment/supplies in the country might be a possible explanation for this TB diagnosis site variations. Nonetheless, the ratio of TB diagnosis sites to a population in the regions is significantly different i.e., 163,317 people per facility (653,266/4) in the Savannah Region, 144,434 people per facility (2,310,939/16) in the Northern Region, 131,789 people per facility (658,946/5) in the North East Region, 75,125 people per facility (901,502/12) in the Upper West Region, 62,741 people per facility (564,668/9) in the Ahafo Region, and 32,531 people per facility (1,301,226/40) in the Upper East Region using the 2020 population and housing census figures [21]. Although this study also revealed a difference in physical accessibility to TB diagnosis sites in the regions, the findings suggest that the residents of Savannah, Northern, North-East, Ahafo, and Upper West regions are the most affected. In most of the districts in these regions, the residents travel beyond 10 km to access a TB diagnostic service. Long distance to facilities providing TB services delays TB diagnosis [23], notification rate [23], treatment initiation, and sometimes return to treatment [9, 10]. Considering that the populations in these regions are mostly rural, farming communities, having limited transportation options, poor road quality and poor electronic connectivity, it is essential to bring TB health services closure to them.

**Fig. 4:**
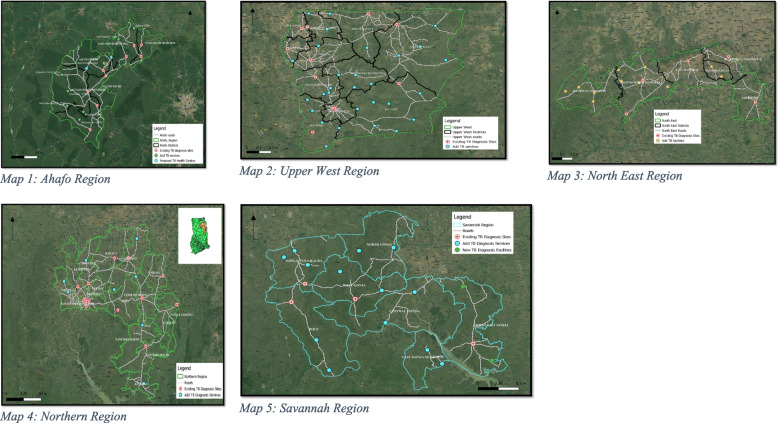
Maps visualising travel time from all locations to the nearest TB diagnostic site in Ahafo, Upper West, North East, Savannah, and Northern Regions of Ghana

To ensure residents in these six regions travel a maximum of 10 km to access a TB diagnostic service, this study has identified 75 priority locations for future placement of TB health services (Fig. 4). However, most of these sites identified are CHPS facilities. We recognize that Tuberculosis diagnosis is beyond the level of CHPS facilities in Ghana. However, to overcome poor accessibility for TB diagnostic facilities with conventional microscopic smear examination and/or Xpert, we suggest the use of newly developed point of care tests, such as urine-based TB tests, AlereLAM or FujiLAM. We further suggest the placement of trained task shifting officers or TB focal persons in CHPS facilities to conduct TB screening, sputum collection, sputum transport to the nearest GeneXpert site, and contact tracing. Moreover, we suggest intensifying targeted community-based interventions aimed at TB case detection and linkage to treatment in communities with poor access to TB care facilities. It is our opinion that these suggestions if considered can help address the low (less than 35%) TB case detection Ghana is currently experiencing.

One major strength of this study is the application of Geographic information systems. Spatial accessibility measures have been demonstrated to be an important policy tool for managing health care delivery and reducing health disparities [24, 25]. However, this study was conducted in six out of the 16 regions in Ghana due to inadequate funding which is a major limitation. We propose a similar study in the remaining ten regions of Ghana a major step toward addressing TB testing challenges in the country. With regards to the available Gene Xpert sites, this study did not check the model types which perhaps could help in future replacement exercises. We encourage future studies to consider this aspect as well.

## Conclusion

In summary, this study's findings suggest limited availability of and poor physical accessibility to TB diagnostic testing sites across five (Upper West, Northern, North-East, Ahafo, and Savannah regions) out of the six regions. This study’s results are dire and could affect Ghana’s progress toward ending TB epidemic by 2030 if not addressed, hence, targeted implementation of additional TB diagnosis sites is needed to reduce travel distances to ≤ 10 km. This study’s analysis showed that a total of 75 additional diagnosis sites will be needed to reduce long distance travels beyond 10 km to facilities providing TB testing services in all six regions. We recommend replication of this study in the remaining 10 regions of Ghana for improvement.

## Supplementary Information

**Additional file 1: Supplementary file 1.** A table showing the distribution of TB diagnosis sites by types in Ahafo, Upper West, North East, Northern, Savannah, and Upper East Regions of Ghana

**Additional file 2: Supplementary file 2.** Maps visualising travel time from all locations to the nearest TB diagnosis site Ahafo, Upper West, North East, Northern, Savannah, and Upper East Regions of Ghana. **Figure S1. **A map visualising travel time from all locations to the nearest TB diagnosis site in the Ahafo Region. **Figure S2. **A map visualising travel time from all locations to the nearest TB diagnostic site in the Upper West Region. **Figure S3. **A map visualising travel time from all locations to the nearest TB diagnostic site in the North-East Region. **Figure S4. **A map visualising travel time from all locations to the nearest TB testing site in the Northern Region. **Figure S5. **A map visualising travel time from all locations to the nearest TB diagnosis site in the Savannah Region. **Figure S6. **A map visualising travel time from all locations to the nearest TB diagnosis site in the Upper East Region.

## Data Availability

Data from this study cannot be shared publicly because it contains sensitive information. All interested researchers/readers/persons who meet the criteria for access to confidential data can access the data set from the first author (Dr. Desmond Kuupiel) via this email: desmondkuupiel98@hotmail.com.
