# Supplementary material for: Geographic availability of and physical accessibility to tuberculosis diagnostic tests in Ghana: a cross-sectional survey
Source: BMC Health Serv Res. 2023 Jul 14;23:755. doi: 10.1186/s12913-023-09755-3 (PMC10347710; doi:10.1186/s12913-023-09755-3)
Supplement: Supplementary file 1 — Additional file 1: Supplementary file 1. A table showing the distribution of TB diagnosis sites by types in Ahafo, Upper West, North East, Northern, Savannah, and Upper East Regions of Ghana [file 12913_2023_9755_MOESM1_ESM.docx]

**Supplementary file 1:** A table showing the distribution of TB diagnosis sites by types in Ahafo, Upper West, North East, Northern, Savannah, and Upper East Regions of Ghana

| **Region (N = 86)** | **Microscopy sites only (n = 56)** | **GeneXpert sites only (n = 7)** | **Microscopy & GeneXpert sites (n =23)** |
| --- | --- | --- | --- |
| **Ahafo Region (n = 9)**  Microscopy sites (n=6)  GeneXpert sites (n=0)  Microscopy & GeneXpert sites (n=3) | John of God Hospital |  | Bechem Government Hospital |
|  | Bomaa Hospital |  | St. Elizabeth Hospital |
|  | Techimantia Hospital |  | Goaso Municipal Hospital |
|  | Keyasi Health Centre |  |  |
|  | Asunafo South District Hospital, |  |  |
|  | Sankore Health Centre |  |  |
| **Upper West Region (n = 12)**  Microscopy sites (n=4)  GeneXpert sites (n=0)  Microscopy & GeneXpert sites (n=8) | St. John Health Centre (Wa East) |  | St. Joseph’s Hospital |
|  | Lambussie Polyclinic |  | Nadowli District Hospital |
|  | Gwollu Hospital |  | Wa West District Hospital |
|  | Wa Urban Health Centre |  | Sissala East District Hospital |
|  |  |  | Lawra Municipal Hospital |
|  |  |  | Upper West Regional Hospital |
|  |  |  | Wa Municipal Hospital |
|  |  |  | St. Theresa’s Hospital (Nandom) |
| **North-East (n = 5)**  Microscopy sites (n=3)  GeneXpert sites (n=2)  Microscopy & GeneXpert sites (n=0) | Chereponi Government Hospital | Baptist Medical Centre (Nalerigu) |  |
|  | Binde Hospital | Walewale District Hospital |  |
|  | Janga Polyclinic |  |  |
|  |  |  |  |
|  |  |  |  |
|  |  |  |  |
|  |  |  |  |
| **Northern Region (n = 16)**  Microscopy sites (n=10)  GeneXpert sites (n=1)  Microscopy & GeneXpert sites (n=5) | Karaga District Hospital | Bimbilla District Hospital | Tamale Central Hospital |
|  | Gushegu District Hospital |  | Tamale Teaching Hospital |
|  | Tatale Polyclinic |  | Assembly of God Hospital |
|  | The King's Medical Centre |  | Yendi District Hospital |
|  | Tamale West Hospital |  | Zabzugu District Hospital |
|  | St. Lucy Polyclinic |  |  |
|  | Tamale Technical University Hospital |  |  |
|  | God Cares Community Hospital |  |  |
|  | Savelugu District Hospital |  |  |
|  | SDA Hospital |  |  |
| **Savannah Region (n = 4)**  Microscopy sites (n=0)  GeneXpert sites (n=4)  Microscopy & GeneXpert sites (n=0) |  | Bole District Hospital |  |
|  |  | Salaga District Hospital |  |
|  |  | Sawla District Hospital |  |
|  |  | Damongo District Hospital |  |
| **Upper East Region (n = 40)**  Microscopy sites (n=33)  GeneXpert sites (n=0)  Microscopy & GeneXpert sites (n=7) | Binaba Health Centre |  | Bawku Presby Hospital |
|  | Sapelliga Health Centre |  | Zebilla Hospital |
|  | Timonde Health Centre |  | Upper East Regional Hospital |
|  | Zuarungu Health Centre |  | Bongo Hospital |
|  | Bongo Soe Health Centre |  | Sandema Hospital |
|  | Namoo Health Centre |  | Garu Health Centre |
|  | Vea Health Centre |  | War Memorial Hospital |
|  | St Theresah Health Centre |  |  |
|  | Siniensi Community Clinic |  |  |
|  | St. Lucas Hospital |  |  |
|  | Fumbisi Health Centre |  |  |
|  | Paga Health Centre |  |  |
|  | Songo Health Centre |  |  |
|  | Worikambo Health Centre |  |  |
|  | Kologo Health Centre |  |  |
|  | Navrongo Health Centre |  |  |
|  | Talensi District Hospital |  |  |
|  | Chiana Health Centre |  |  |
|  | Kandiga Health Centre |  |  |
|  | Kayoro Health Centre |  |  |
|  | Nakolo Health Centre |  |  |
|  | Martyrs of Uganda Health Centre |  |  |
|  | Duusi Health Centre |  |  |
|  | Kongo Logre Clinic |  |  |
|  | Nangodi Health Centre |  |  |
|  | Kulungungu Health Centre |  |  |
|  | Pusiga Health Centre, |  |  |
|  | Datoku Health Centre |  |  |
|  | Namolgo Clinic |  |  |
|  | Pwalugu Health Center |  |  |
|  | Winkongo Health Centre |  |  |
|  | Bugri Health Centre |  |  |
|  | Woriyanga Health Centre |  |  |
