# Supplementary material for: Geographic availability of and physical accessibility to tuberculosis diagnostic tests in Ghana: a cross-sectional survey
Source: BMC Health Serv Res. 2023 Jul 14;23:755. doi: 10.1186/s12913-023-09755-3 (PMC10347710; doi:10.1186/s12913-023-09755-3)
Supplement: Supplementary file 2 — Additional file 2: Supplementary file 2. Maps visualising travel time from all locations to the nearest TB diagnosis site Ahafo, Upper West, North East, Northern, Savannah, and Upper East Regions of Ghana. Figure S1. A map visualising travel time from all locations to the nearest TB diagnosis site in the Ahafo Region. Figure S2. A map visualising travel time from all locations to the nearest TB diagnostic site in the Upper West Region. Figure S3. A map visualising travel time from all locations to the nearest TB diagnostic site in the North-East Region. Figure S4. A map visualising travel time from all locations to the nearest TB testing site in the Northern Region. Figure S5. A map visualising travel time from all locations to the nearest TB diagnosis site in the Savannah Region. Figure S6. A map visualising travel time from all locations to the nearest TB diagnosis site in the Upper East Region. [file 12913_2023_9755_MOESM2_ESM.docx]

**Supplementary file 2:** Maps visualising travel time from all locations to the nearest TB diagnosis site Ahafo, Upper West, North East, Northern, Savannah, and Upper East Regions of Ghana


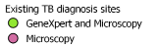


**
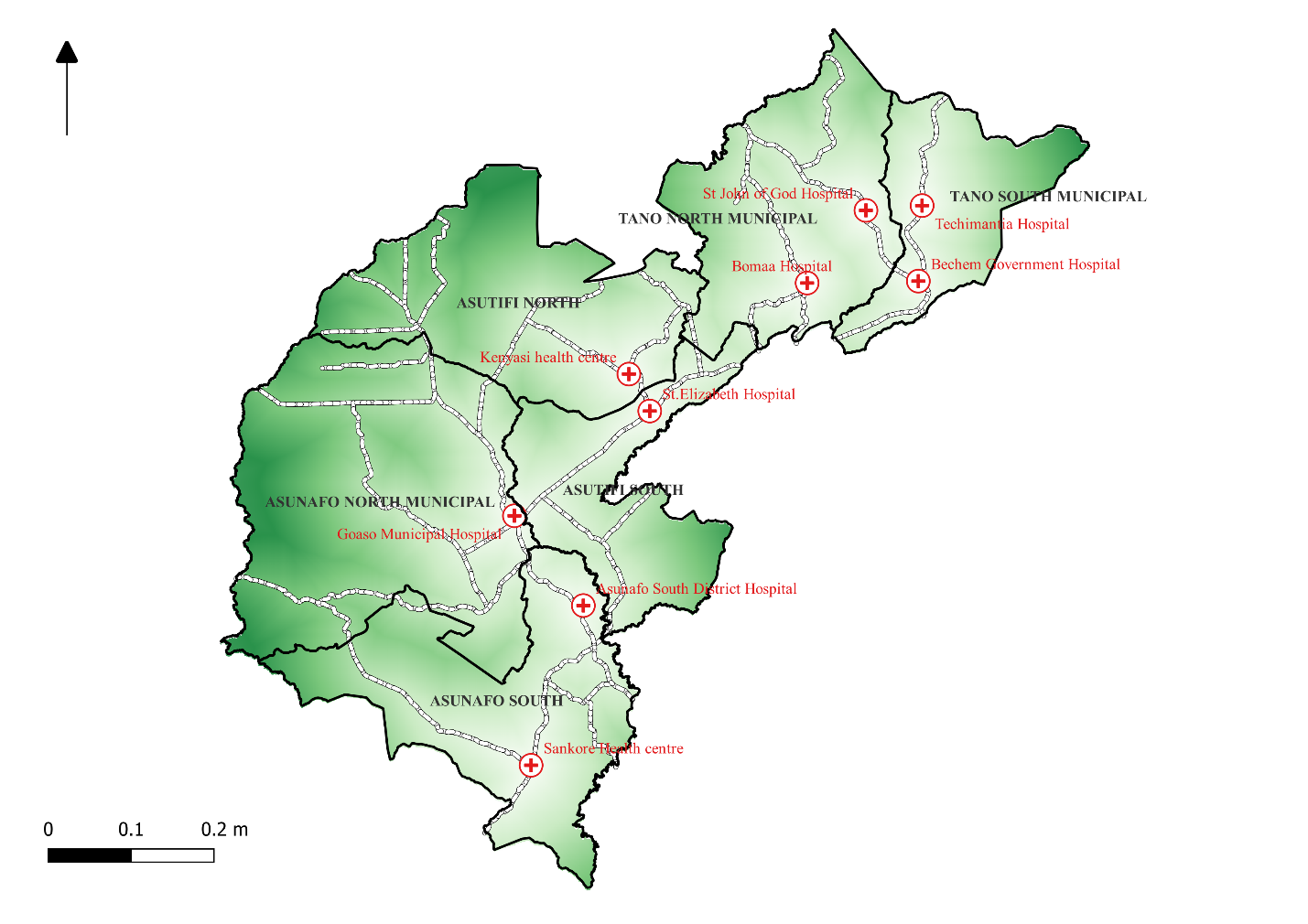
****Figure S1:** A map visualising travel time from all locations to the nearest TB diagnosis site in the Ahafo Region


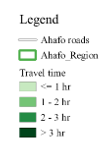


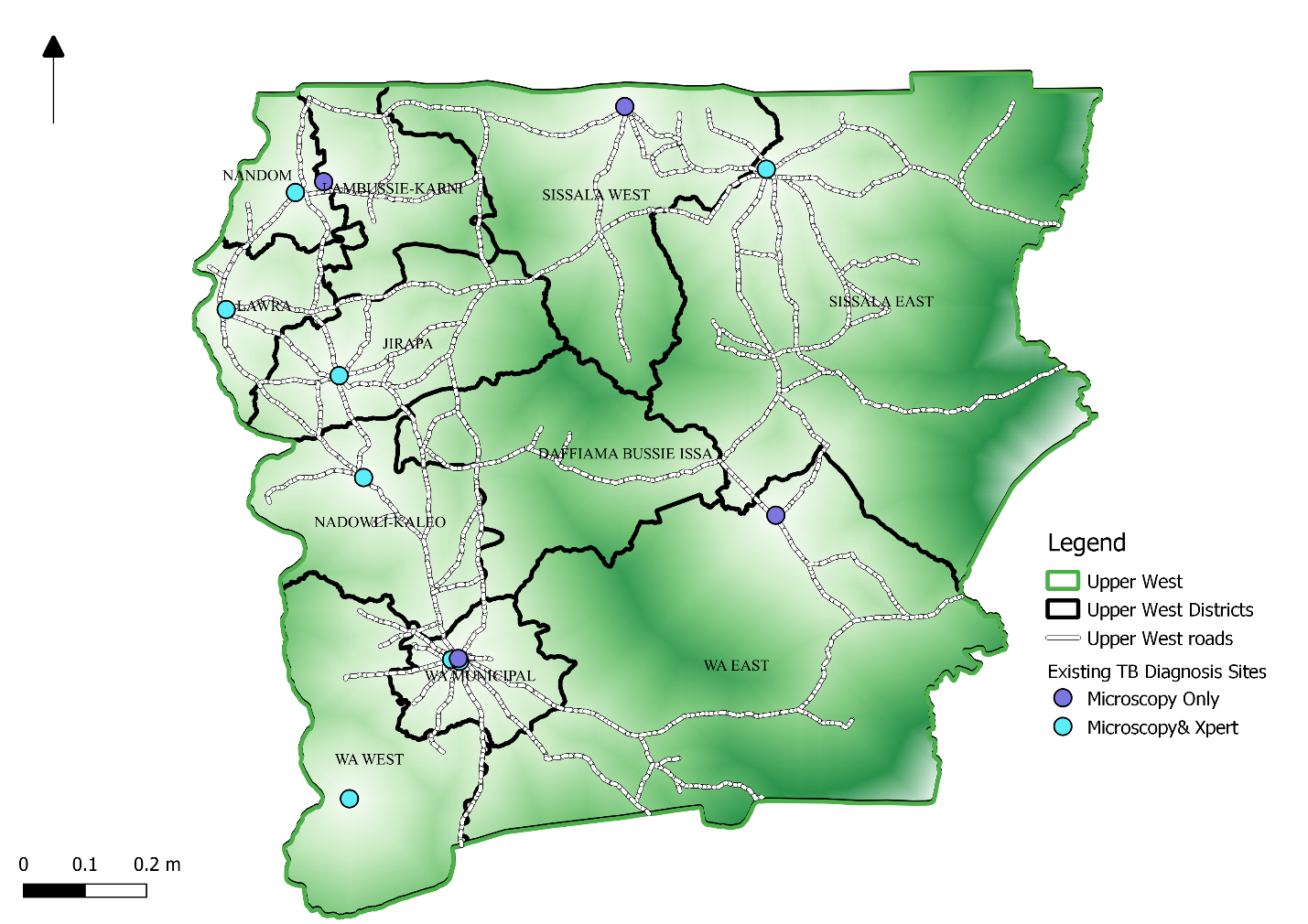
**Figure S2:** A map visualising travel time from all locations to the nearest TB diagnostic site in the Upper West Region


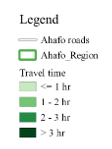


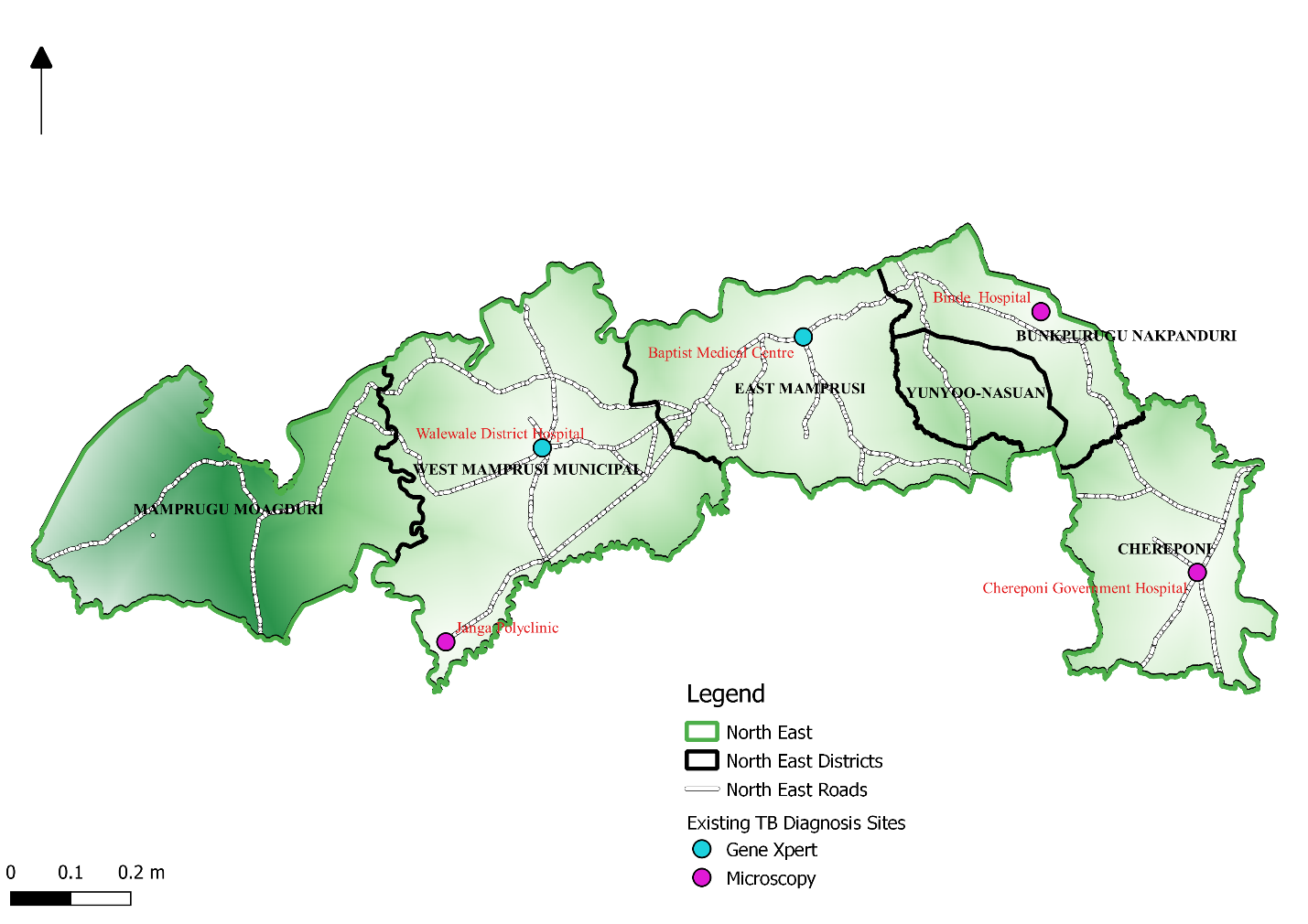
**Figure S3:** A map visualising travel time from all locations to the nearest TB diagnostic site in the North-East Region


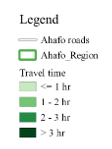


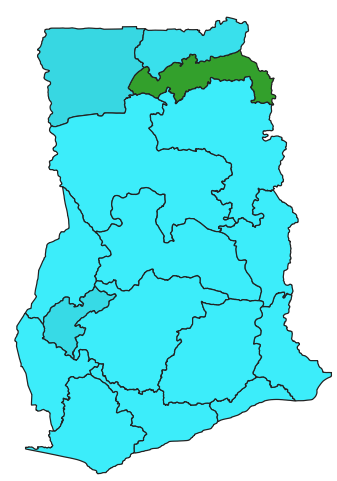


**
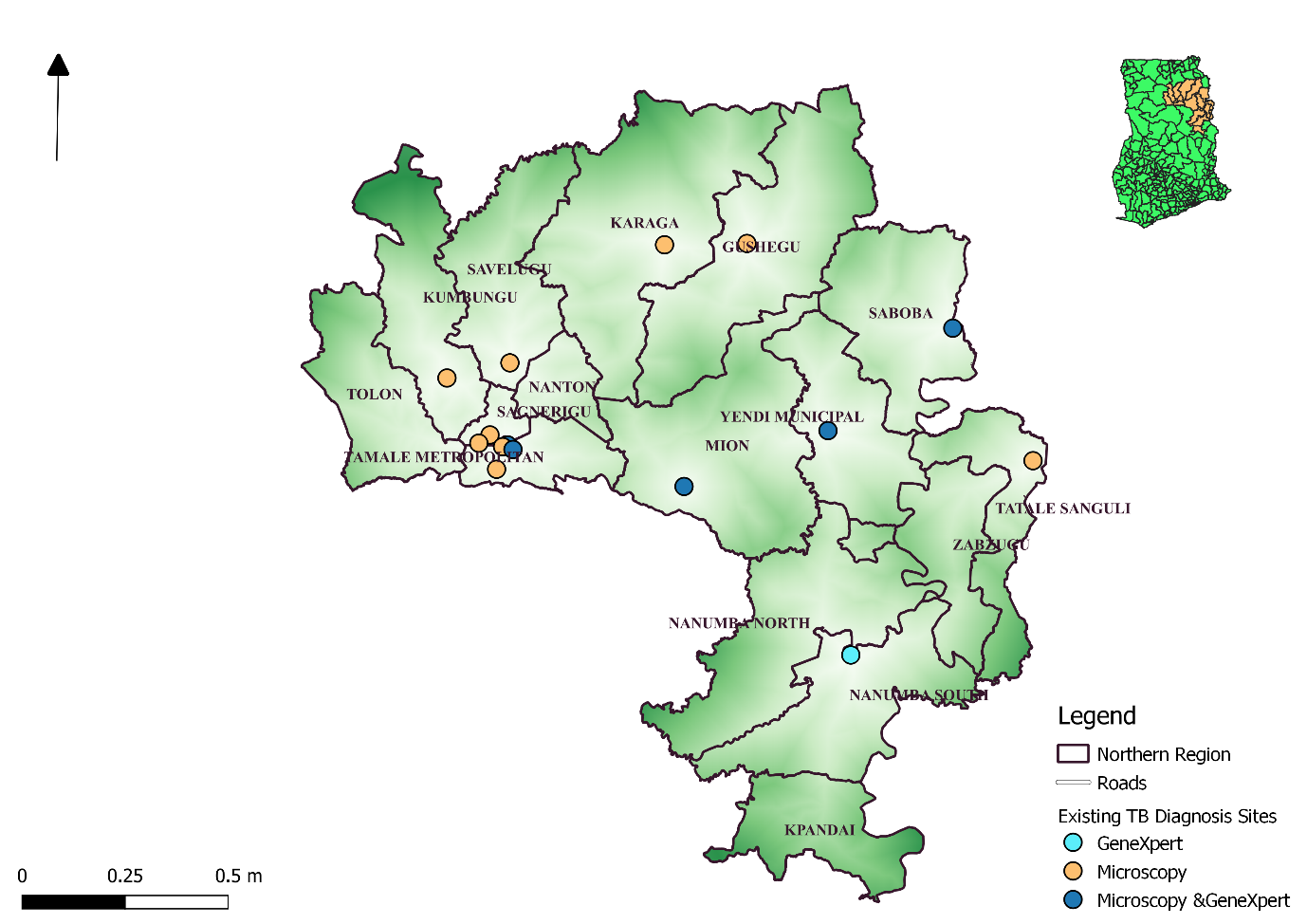
Figure S4:** A map visualising travel time from all locations to the nearest TB testing site in the Northern Region


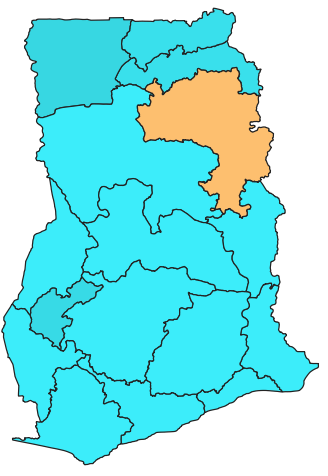

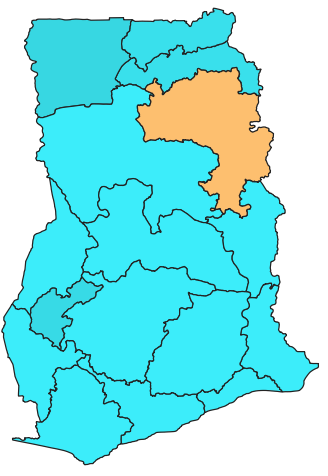


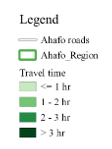


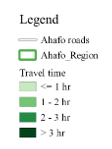


**
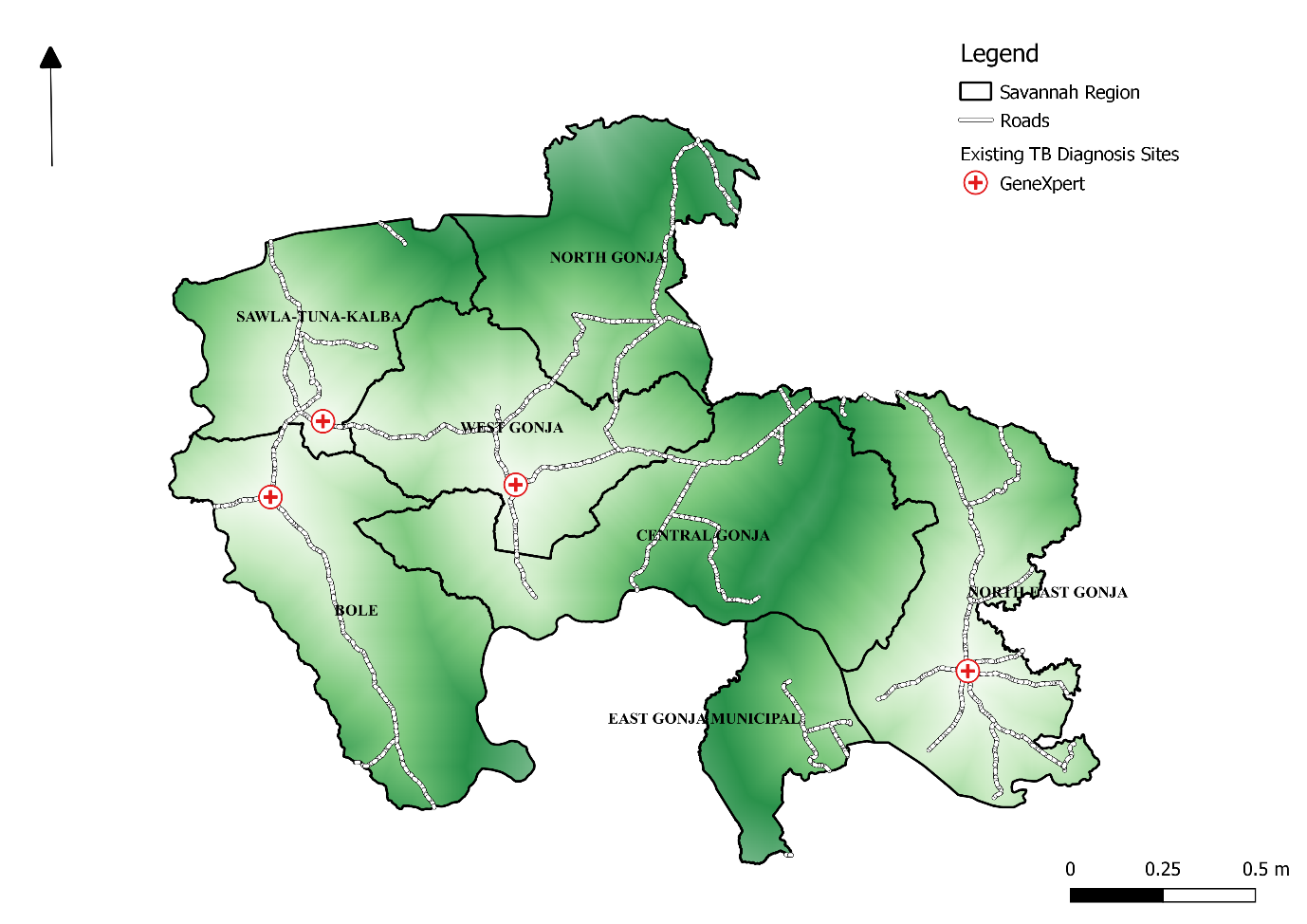
**

**Figure S5:** A map visualising travel time from all locations to the nearest TB diagnosis site in the Savannah Region

**
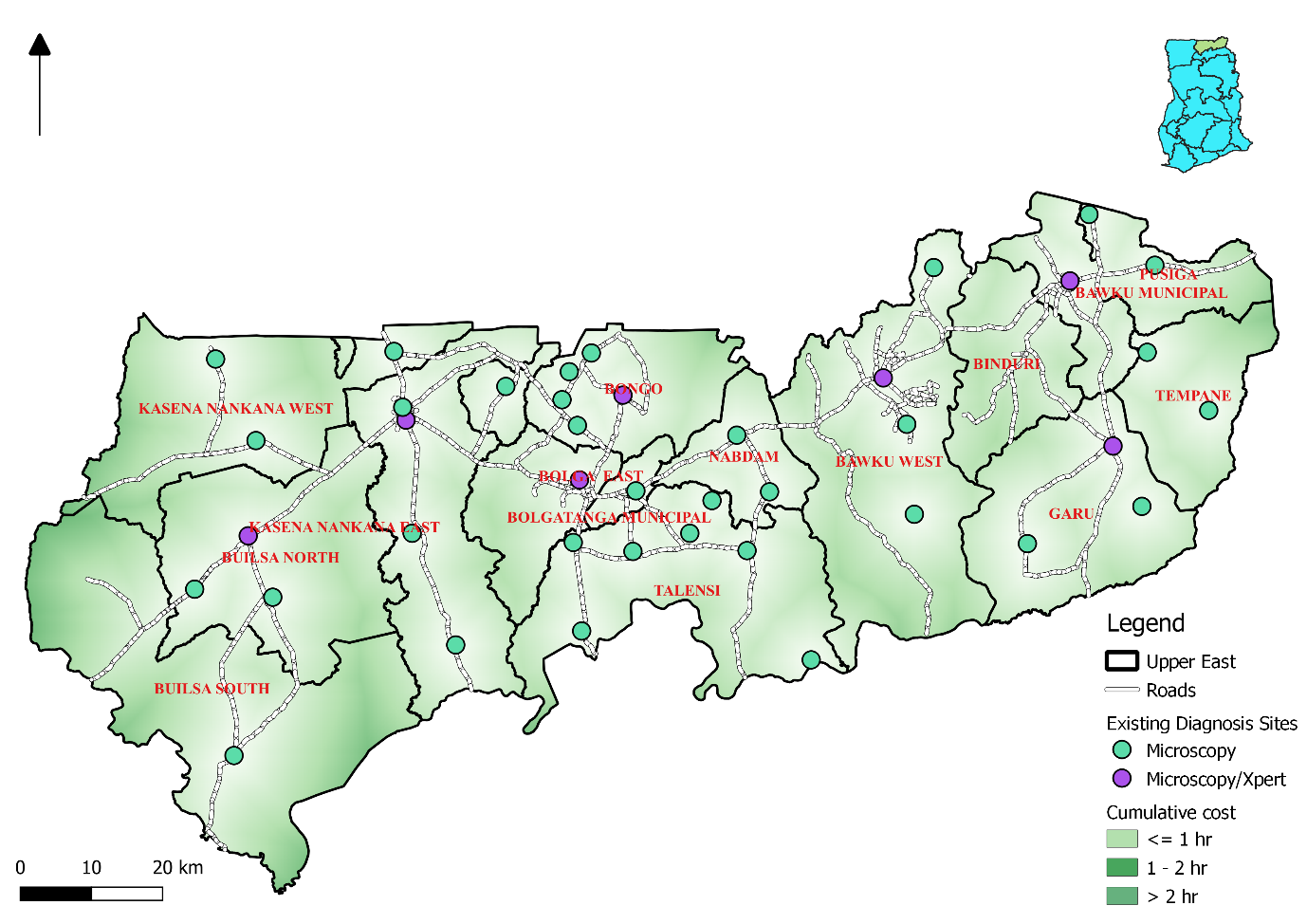
**

**Figure S6:** A map visualising travel time from all locations to the nearest TB diagnosis site in the Upper East Region
